# Evaluation of Follow-up Colposcopy Procedures After Abnormal Cervical Screening Result Across a Statewide Study in Mississippi

**DOI:** 10.21203/rs.3.rs-3943646/v1

**Published:** 2024-02-13

**Authors:** Sydney Reaves, Katherine C. Hall, Mary W. Stewart, Nicolas Wentzensen, Christina Ferrell, Carolann Risley, Jimmie Wells, Rhonda Rives, Fajada Bobo, Jon Daniels, Kathy Farrington, Jody C. Morgan, Megan A. Clarke

**Affiliations:** University of Mississippi Medical Center, School of Nursing; University of Mississippi Medical Center, School of Nursing; Mississippi State University, School of Nursing; National Cancer Institute; University of Mississippi Medical Center, School of Nursing; University of Mississippi Medical Center, Cancer Research Institute; University of Mississippi Medical Center, School of Nursing; Mississippi Baptist Medical Center, Department of Pathology; University of Mississippi Medical Center, Department of Pathology; Mississippi State Department of Health; Mississippi State Department of Health; University of Mississippi Medical Center, School of Medicine; National Cancer Institute

## Abstract

**Purpose:**

Cervical screening is used to detect and treat precancers to prevent invasive cancers. However, successful prevention also requires adequate follow-up and treatment of individuals with abnormal screening results. The aim was to investigate demographics, clinical characteristics, and follow-up status for individuals needing colposcopy after an abnormal screening result.

**Methods:**

The STRIDES (**St**udying **R**isk to **I**mprove **D**ispariti**ES**) cohort comprises individuals undergoing cervical cancer screening and management at a Mississippi Health Department or University of Mississippi clinic. Follow-up status, demographics, and clinical data were assessed from electronic health records and, if necessary, patient navigation on individuals identified as needing a colposcopy after an abnormal screening.

**Results:**

Of the 1,458 individuals requiring colposcopy, 43.0% had the procedure within 4 months, 16.4% had a delayed procedure, and 39.5% had no documented follow-up, with significant predictors of follow-up identified as age and cytology diagnosis. Based on age, individuals 30 + were more likely to follow up with a colposcopy compared to individuals < 30 years (49% and 38.7%, respectively; p < .001). Individuals with cytology diagnoses of LSIL (52.9%), ASC-H (51.4%), and HSIL (62.3%) had higher percentages of adherence to follow-up colposcopy guidelines (p < .001).

**Conclusion:**

Despite high cervical cancer screening rates among Mississippians, a substantial portion did not have adequate next-step intervention. However, it is encouraging that highest risk individuals were more likely to have a colposcopy. Regardless, continuing to understand the underlying causes for incomplete follow-up is crucial for timely secondary targeted interventions to reduce cervical cancer burden, promote awareness, and improve health outcomes.

## INTRODUCTION

Cervical cancer (CC), caused by persistent infection with carcinogenic human papillomavirus (HPV), is one of the few preventable cancers. Dramatic reductions in incidence and mortality rates have been seen in the United States (U.S.) over the last several decades due to the utilization of the Papanicolaou (Pap) smear screening. With the more recent advent of the HPVvaccine and the increased use of HPV-based testing, either alone or with Pap cytology (co-testing) as primary screening approaches, even more reductions in CC incidence and mortality are expected [1]. However, despite availability of primary and secondary prevention approaches, over 14,000 new CC cases and more than 4,000 CC deaths occurred in the U.S. in 2022 alone [2]. More than half of new CC diagnoses in the U.S. occur in individuals who are never screened or who are under-screened [3]. Beyond screening, secondary prevention of CC requires effective and timely follow-up with diagnostic colposcopy and targeted biopsies of screen-positive individuals and treatment of cervical precancers if detected. Without these steps, CC prevention fails, and screened individuals remain at elevated risk for CC [4].

Within the U.S., there are known geographic disparities with respect to cervical cancer incidence and mortality. Mississippi ranks among the top five states in the nation for CC incidence and mortality, despite having among the highest CC screening rates [5–6]. This scenario suggests that high rates of cervical cancer in Mississippi may, in part, be due to a lack of diagnostic follow-up and treatment among screened individuals. We sought to evaluate patient demographics, clinical characteristics, and adherence to follow-up colposcopy care for individuals with an abnormal CC screening results within a large, statewide cohort of patients undergoing screening in Mississippi.

## METHODS

### Study Population

The National Cancer Institute (NCI), Mississippi State Department of Health (MSDH), and the School of Nursing at the University of Mississippi Medical Center (UMMC) developed the STRIDES – **St**udying **R**isk to **I**mprove **D**ispariti**ES** study in 2019. STRIDES represents a statewide cohort of individuals undergoing cervical cancer screening and management at UMMC and MSDH. A detailed description of the STRIDES study design is available elsewhere (5).

### Procedures

For this study, nested within the STRIDES cohort, we identified individuals with the following criteria: screened from January 2018 through August 2021 at an MSDH clinic and had an abnormal screening result with an indication for colposcopy based on the 2012 ASCCP management guidelines [7]. Once the sample was obtained, we performed chart reviews of electronic health records recurrently (EHR; i.e., the MSDH histology file) through September 2022 to search for documentation indicating receipt of any cervical cancer follow-up care by an MSDH or outside provider. Receipt of follow-up care was defined according to the presence of histology resulting from a cervical biopsy in the EHR and extracted for our records. After chart reviews any individual found to be lacking follow-up documentation in the EHR were then provided to a team of patient navigators, who worked with MSDH clinic staff to contact individuals and schedule follow-up appointments.

### Study variables

MSDH screening procedures were based on the 2012 ASCCP guidelines and included cytology with HPV triage in the presence of atypical squamous cells of undetermined significance (ASC-US) for patients aged 21–29 and co-testing for patients aged 30+. Cytological diagnoses were obtained from the EHR and classified according to the Bethesda System as NILM, negative for intraepithelial lesion or malignancy; ASC-US; LSIL, low-grade intraepithelial lesion; ASC-H, atypical squamous cells cannot exclude high grade; or HSIL, high-grade intraepithelial lesion. HPV testing was performed using cobas4800 on the ThinPrep sample collected during the Pap smear screening with results reported in the EHR. HPV genotypes were reported as HPV type 16, HPV type 18, and pooled HPV other high-risk types (HR12): 31, 33, 35, 39, 45, 51, 52, 56, 58, 59, 66, and 68.

At the time of the CC screening visit, we collected sociodemographic characteristics from the EHR. Age was categorized as < 30 years and ≥ 30 years. Race and ethnicity were categorized as non-Hispanic White, non-Hispanic Black, all Hispanic, other (combined Asian or Native American ethnicities), and unknown. Smoking was recorded as never, former, current, and unknown/missing. Body mass index (BMI; kg/m^2^) was categorized according to standard definitions as < 25 (underweight/healthy weight), 25 to < 30 (overweight), 30 to < 35 (class I obesity), and >/=35 (class II obesity).

The primary outcome was adherence to follow-up recommendations for a colposcopy based on the guidelines used at the time of the screening event. Follow-up status was determined based on the evaluation of any follow-up documentation to determine if individuals returned for their follow-up colposcopy after an abnormal cervical cancer screening result. Individuals were placed into three groups: 1. Adherent to Follow-Up with Colposcopy (on time) = individuals who returned to their provider and had a colposcopy within the recommended 4 month timeframe; 2. Adherent to Follow-up with Colposcopy (delayed) = Individuals who returned to their provider and had a colposcopy outside of the recommended 4 month timeframe; 3. Non-Adherent to Follow-Up Recommendations = Individuals who did not return for the recommended follow-up colposcopy by September 2022. The non-adherent group also includes individuals who may have returned for follow-up but received a co-test instead of a colposcopy.

### Data Analysis

We assessed patient demographics, clinical characteristics, and adherence to follow-up recommendations using descriptive statistics and reported means and standard deviations for continuous variables, as well as frequencies and percentages for categorical variables. We compared characteristics of individuals who were adherent (on time), adherent (delayed), and non-adherent to follow-up care using chi-square analysis. We estimated the odd ratios (ORs) and 95% confidence intervals (CIs) for associations of individual and clinical characteristics with follow-up using multivariable logistic regression analyses to predict follow-up vs. no follow-up within the sample. We used Kaplan-Meier methods to assess receipt of colposcopy over time among individuals with abnormal screening results and compared the survival curves based on age and cytology using log-rank tests. Statistical analyses were performed using IBM SPSS Statistics version 28 (IBM Inc., Armonk, NY, USA).

## RESULTS

Between 2018–2021 the STRIDES study had a population size of 32,735 individuals undergoing screening within Mississippi’s public health system, with 20,792 individuals being seen at a MSDH clinic. We identified 1,458 MSDH individuals that had an abnormal result indicating need for a colposcopy based on the 2012 ASCCP guidelines. Characteristics of these individuals by follow-up status are shown in Table 1 with a full breakdown of cytology and HPV results shown in Supplemental Table S1 and S2. A total of 627 individuals (43.0%) with an abnormal screening result had a follow-up colposcopy procedure documented within 4 months (on time). There were 239 (16.4%) individuals that had follow-up documentation of a colposcopy procedure, but outside of the recommended four-month period with a delayed mean of 15.6 months (SD = 12.91) and a maximum of 53 months seen. Of the 592 individuals (40.6%) identified as not adherent to the follow-up guidelines, 264 (18.1%) had documentation of a return visit where they received an additional co-test instead of the recommended colposcopy. Characteristics of individuals who returned for a repeat co-test compared to those who did have follow-up care documented are shown in Supplemental Table S3.

The follow-up outcome groups had significant differences noted between adherence to follow-up recommendations by age, race/ethnicity, and cytology diagnosis shown in Table 1. Regarding age, 49.0% of individuals ≥ 30 years of age compared to 38.7% of individuals <30 years of age were adherent to follow-up guidelines *(p* <.001). Regarding race and ethnicity *(p* = .003), individuals who were Hispanic had the highest percentage (63.3%) of having follow-up care based on recommendations. Individuals listed as Other had the highest percentage (48.2%) of not having documented follow-up care. When looking at cytology diagnosis (*p* <.001), individuals with a low-risk cytology diagnosis such as ASC-US (53.6%) and LSIL (31.9%) were less likely to receive follow-up compared to individuals with a more severe cytology diagnosis, such as ASC-H (25.0%) and HSIL (21.6%). Individuals with an HPV-positive NILM diagnosis (44.2%) also had a higher percentage of being less likely to receive follow-up. There was no difference in follow-up outcomes by BMI category (*p* = 0.105) or smoking status (*p* = 0.216).

We conducted multivariate logistic regression analyses to investigate factors associated with follow-up care, shown in Table 2. Overall, age (per one-year increase) was positively associated with an increased likelihood of following up with a colposcopy recommendation (OR = 1.07; 95% CI = 1.05, 1.09). When further stratified by age, younger individuals (<30 years) observed a 36% increase (OR = 1.36; 95% CI = 1.26, 1.47; *p* <.001) in the likelihood of following up with a colposcopy with every one-year increase in age. However, in individuals ≥30 years old, the same pattern of increased likelihood of colposcopy follow-up was not significantly identified. (OR = 1.01; 95% CI = 0.98, 1.47; *p* = .556). Cytology diagnoses were also associated with increased likelihood of completing a colposcopy follow-up in the various regression models. When comparing individuals with a NILM cytology result to the full sample, those with LSIL, ASC-H, and HSIL were more likely to follow up with colposcopy, (OR = 2.67, 3.9, and 5.37, respectively; p<.001). Similar findings among cytological diagnoses were observed when stratified by age groups.

Supplemental tables S1 and S2 show the distribution of cytology screening results by HPV genotype. Among the 617 individuals ≥ 30 years of age, 90.4% (n = 553) of the cytology results had an associated HPV test result, with 96.2% (n = 532) of those screening HPV positive. Among individuals <30 with ASC-US cytology diagnosis, HPV testing was completed on 99.8% (n = 428) individuals. HPV Other HR12 was the most common result among screened-positive HPV genotypes for the sample of individuals <30 (n=370, 86.2%) whereas HPV 16/18 was the most frequent subtype among HPV screened positive individuals ≥ 30 (n=281, 45.9%). Among individuals with HPV 16/18, both LSIL (OR = 4.00; 95% CI = 1.25, 12.79; *p* = .019) and ASC-H/HSIL (OR = 2.07, 95% CI = .93, 4.59, *p* = .074) cytology diagnoses results were more likely to go to colposcopy, whereas among Other HR12 only the cytology diagnosis of ASC-H/HSIL (OR = 4.16; 95% CI = 1.27, 13.67; *p* = .019) were more likely to receive a colposcopy for follow-up care (data not shown).

**Figure 1:**
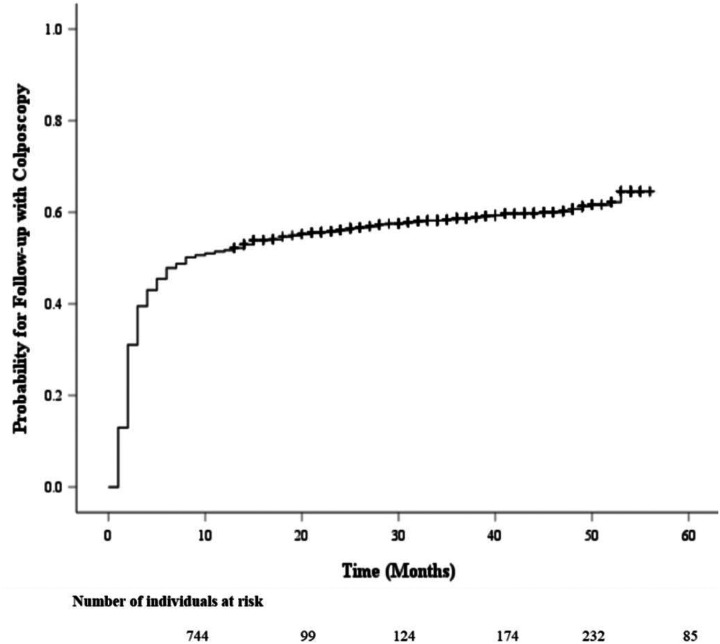
The Table of Follow-up with a Colposcopy Time

**Figure 2:**
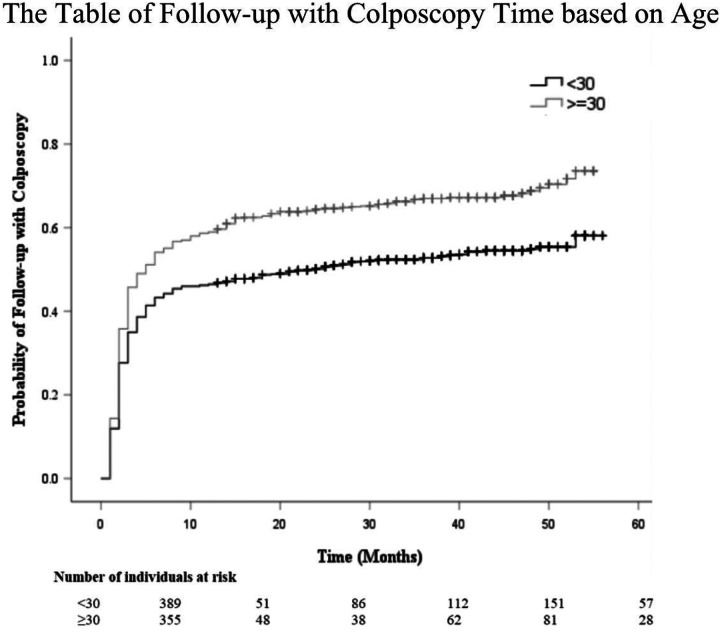
The Table of Follow-up with Colposcopy Time based on Age

**Figure 3:**
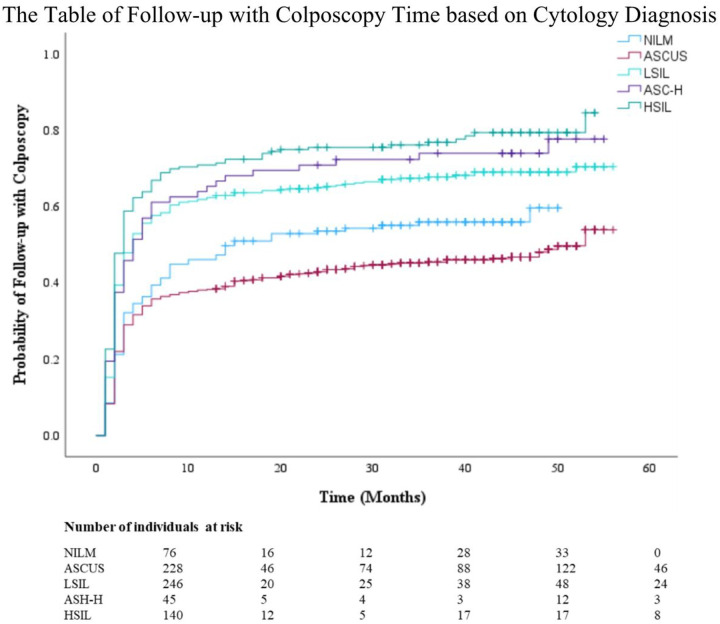
The Table of Follow-up with Colposcopy Time based on Cytology Diagnosis

The Kaplan-Meier survival curve probability for follow-up care with a colposcopy after an abnormal screening result is displayed in Figure 1. Most individuals who underwent a colposcopy did so within 12 months (0.5 probability by 8 months), then continued to increase at a slower rate up to 0.6 at year 5. Figure 2 provides a comparison of colposcopy probability between different age groups. The curve identifies individuals aged 30 years and older had a significantly higher probability for follow-up colposcopy at nearly 0.6 after one year and 0.7 at year 5 compared to individuals aged less than 30 years (*p* <.001). Figure 3 represents a comparison of follow-up colposcopy probability among cytological diagnoses with significant differences noted among the five cytology diagnoses (*p* <.001). Individuals with a higher risk cytology diagnosis of HSIL or ACSUS had a higher probability of following up with a colposcopy (0.7 and 0.6 after one year, respectively) compared to other diagnoses.

## DISCUSSION

Colposcopy is a critical secondary preventative diagnostic procedure used to evaluate cervical abnormalities detected during routine screening, and the adherence to guidelines ensures standardized and evidence-based management recommendations [7]. In our study, we evaluated patient demographics and clinical characteristics in relation to follow-up status among individuals needing a colposcopy based on ASCCP guidelines after an abnormal cervical cancer screening result. We identified 1,458 individuals screened at a MSDH clinic, between 2018–2021, who needed colposcopy after their abnormal screening result. A majority (43.0%) of individuals with an abnormal screening result had a follow-up colposcopy procedure within 4 months (on time). Adherence to guidelines significantly differed between age, race/ethnicity, and cytology diagnosis. The likelihood of follow-up colposcopy adherence increased with age and with increased cytology diagnosis severity (i.e., LSIL, ASC-H, and HSIL).

Further categorization of timely adherence to the recommended colposcopy was categorized into three groups and a significant concern identified was that 39.5% of the individuals were not adherent to the guidelines for follow-up care. Further, 328 (55.4% of the 39.5% not adherent) individuals had no documented follow-up of any kind. Thus, challenges exist to ensure that patients with abnormal cervical cancer screening results persist in receiving necessary follow-up care. Previous studies evaluating adherence to colposcopy follow-up found that the majority of individuals fell within the adherent (on-time) category, but 42.3% were not adherent, which is consistent with our findings [8]. Together, these findings identify the need to continue to explore challenges surrounding timely and appropriate cervical cancer follow-up care, particularly among Mississippi’s vulnerable populations.

Martinez-Gutierrez et al. (2023) recently published a systematic review of 26 studies including 265,041 individuals from high-income countries who required follow-up after a cervical cancer screening. Over 40 factors were used to define inadequate follow-up, with younger, less educated, and lower socioeconomic status (SES) being associated with inadequate follow-up [9]. While socioeconomic variables were not included within our study analysis, this study is best interpreted within the context of the patient population seeking care at MSDH clinics. MSDH is the primary source of healthcare for Mississippians that are underinsured/ uninsured and that fall within the lower ranges of SES [10]. However, the problem of inadequate follow-up for patients at risk for cervical cancer is not limited to socioeconomic status.

Age was also found to be a significant factor in follow-up colposcopy care [9]. In our study, 46.5% of individuals who are < 30 years old were non-adherent with follow-up colposcopy care but an increased likelihood of colposcopy with every one year of increased age up to 30 was noted. In Sharp et al. (2012), who published a prospective cohort study including 2,213 individuals needing colposcopy, age was also significantly associated with not returning to the clinic for follow-up colposcopy care. In this report, and like our findings, individuals < 30 years old were found at the highest risk of non-adherence with risk of non-adherence reducing by one-third in patients aged 30–39 years and by two-thirds in those aged 40–59 [11].

Our study found that increased likelihood of colposcopy follow-up care increased with cytology severity. Literature has found that colposcopy diagnosis tends to correlate well with high-grade abnormalities but is less efficient for women with lower-level cytological abnormalities [12]. Studies show individuals with lower cytological diagnosis (ASCUS or NILM but HPV positive) are less likely to receive a colposcopy as their follow up care as some providers and/or patients may prefer conservative management [13]. Our study’s findings align in that individuals with lower grade cytology were less likely to receive a colposcopy yet account for 73.9% of the 264 individuals who performed a follow-up repeat co-test instead. Perkins et al. (2021) study found that adherence to follow-up colposcopy guidelines, within 6 months, had the following rank order: high-grade cytology (defined as HSIL & ASCH) > low-grade cytology (LSIL, ASCUS) > HPV-positive NILM cytology [14]. This same pattern was identified within our study with 62.3% of HSIL diagnosis and 51.4% of ASC-H diagnosis being adherent (on time).

### Strength/ Limitations:

Mississippi has a racially and ethnically diverse population, and one notable aspect of our study is its inclusion of a substantial percentage of African American individuals and those residing in rural areas who have been underrepresented within cervical cancer research despite having a disproportionate burden of the disease and mortality [5]. This diversity is essential to display as tailored screening strategies may result from a clearer understanding of any differences among groups [15]. Additionally, all individuals who went to a MSDH clinic for cervical cancer screening and follow-up during the data collection period were included in the biorepository. Other than individuals sent to outside providers, all specimens were routed through a single cytopathology laboratory, allowing for consistent and complete ascertainment of data from patients who underwent care at MSDH. Further, we performed thorough chart reviews to collect any information from patients who sought follow-up care by an outside provider.

Even with the valuable insights obtained from our study, a few limitations should be acknowledged. First, this data was obtained from electronic health records. Despite best efforts, missing or incomplete information is possible. Additionally, the study was conducted within the context of the Mississippi public health care population, and the findings might not be directly generalizable to other regions/populations.

### Future Implications:

There are multiple social and structural barriers identified that disproportionately impact underserved populations, like the population seen in Mississippi’s public health system [16–17]. Further research is needed to explore these barriers and facilitators that impact individual follow-up to cervical cancer care in Mississippi to address identified disparities and inequalities [18–19]. Additionally, understanding of the social and structural factors impacting adherence to follow-up recommendations can inform the development of future targeted interventions and patient-centered strategies. Engaging patient navigators and leveraging community resources might be crucial in facilitating and supporting individuals in their follow-up care [20–23]. In relation to the 18.48% (of the total n = 1,458) of individuals who returned for a repeat co-test instead of the recommended colposcopy, further research is necessary to understand patient’s perspective. Therefore, educational campaigns and outreach initiatives can help raise awareness about the importance of timely colposcopy and help eliminate misconceptions that might prevent individuals from seeking appropriate care [24–25].

In conclusion, our study shows that less than 50% of patients attending MSDH clinics with an abnormal screening result underwent timely and/or appropriate follow-up care. Adherence to management guidelines is essential for accurate diagnosis and appropriate management of cervical abnormalities, which will ultimately contribute to the reduction of cervical cancer incidence and mortality. Identifying and addressing barriers to timely follow-up care will be crucial for reducing the burden of cervical cancer and improving health outcomes in underserved populations, like Mississippi. Our findings call for collaborative efforts among healthcare providers, policymakers, and communities to implement targeted interventions to bridge the gaps in cervical cancer care and strive for better health equity and improved patient outcomes.

## Figures and Tables

**Table 1:** Frequencies and Chi-Square Results for Follow-up Outcomes Among Individuals Following Abnormal Cervical Cancer Screening

	Adherent: Follow-Up with Colposcopy (on time) (N=627)		Adherent: Follow-Up with Colposcopy (delayed) (N=239)		Non-Adherent: No Follow-Up (N=592)		*P* value
	N	row%	N	row%	N	row%	
Age							<.001
<30 years old	327	38.7%	126	14.9%	394	46.5%	
≥30 years old	300	49.0%	113	18.5%	199	32.5%	
Race							.003
Non-Hispanic White	156	43.7	57	16.0	144	40.3	
Non-Hispanic Black	339	41.4	143	17.5	336	41.1	
All Hispanic	57	63.3	12	13.3	21	23.3	
Other	40	36.4	17	15.5	53	48.2	
BMI							.105
<25	166	39.8	68	16.3	183	43.9	
25-<30	125	38.2	60	18.3	142	43.4	
30-<35	135	50.0	40	14.8	95	35.2	
35+	147	43.5	55	16.3	136	40.2	
Smoking							.216
Never Smoker	353	43.4	125	15.4	336	41.3	
Former	84	49.1	29	17.0	58	33.9	
Current	190	40.3	85	18.0	197	41.7	
Cytology Diagnosis							<.001
NILM^a^	57	34.5	35	21.2	73	44.2	
ASC-US	191	31.6	89	14.7	324	53.6	
LSIL	212	52.9	61	15.2	128	31.9	
ASC-H	37	51.4	17	23.6	18	25.0	
HSIL	124	62.3	32	16.1	43	21.6	

*Note*. Abbreviations: ASC-H, atypical squamous cells cannot exclude high grade; ASC-US, atypical squamous cells of undetermined significance; HSIL, high-grade intraepithelial lesion; LSIL, low-grade intraepithelial lesion; NILM, negative for intraepithelial lesion or malignancy; Pap, Papanicolaou test.

a NILM diagnoses were HPV 16, HPV 18, or HPV 12 HR other positive, indicating the need for further management with a colposcopy based on ASCCP 2012 guidelines.

**Table 2:** Regression Results for Follow-up with a Colposcopy Based on Total Sample (N=1458) and Age-Stratified for < 30 (N=846) and ≥ 30 (N=612)

Variable	Model 1: Full Sample				Model 2: < 30-Year-Old				Model 3: ≥ 30-Year-Old			
	*OR*	95% CI		*p*	*OR*	95% CI		*p*	*OR*	95% CI		*p*
Age	1.07	[1.05,	1.09]	<.001	1.36	[1.26,	1.47]	<.001	1.01	[0.98,	1.04]	.556
Race												
Non-Hispanic White	Ref			.203	Ref			.665	Ref			.737
Non-Hispanic Black	1.17	[0.86,	1.57]	.319	1.13	[0.74,	1.71]	.579	1.14	[0.72,	1.80]	.587
All Hispanic	1.72	[0.93,	3.18]	.083	1.69	[0.68,	4.21]	.260	1.17	[0.49,	2.75]	.727
Other	0.88	[0.55,	1.43]	.611	0.96	[0.51,	1.81]	.901	0.75	[0.33,	1.71]	.490
BMI												
<25	Ref			.312	Ref			.709	Ref			.792
25-<30	0.87	[0.63,	1.21]	.410	0.79	[0.51,	1.24]	.314	0.99	[0.58,	1.71]	.995
30-<35	1.19	[0.84,	1.68]	.338	1.02	[0.62,	1.66]	.952	1.27	[0.74,	2.18]	.390
35+	1.14	[0.83,	1.58]	.417	1.01	[0.65,	1.57]	.953	1.03	[0.60,	1.77]	.907
Smoking												
Never Smoker	Ref			.227	Ref			.624	Ref			.064
Current	0.86	[0.65,	1.14]	.298	0.97	[0.66,	1.44]	.891	0.62	[0.40,	0.96]	.034
Former	1.22	[0.82,	1.81]	.320	1.28	[0.74,	2.19]	.378	1.07	[0.56,	2.05]	.847
Cytology Diagnosis												
NILM	Ref			<.001	–	–	–	–	Ref			.005
ASC-US	1.18	[0.77,	1.81]	.451	Ref			<.001	1.59	[0.98,	2.58]	0.60
LSIL	2.67	[1.72,	4.14]	<.001	1.34	[.893,	2.01]	.157	2.78	[1.60,	4.81]	<.001
ASC-H	3.90	[1.91,	7.97]	<.001	6.60	[2.79,	15.62]	<.001	2.06	[0.80,	5.31]	.134
HSIL	5.37	[3.13,	9.20]	<.001	8.95	[4.97,	16.12]	<.001	2.48	[1.32,	4.68]	.005
Atypical	1.12	[0.31,	4.10]	.865	–	–	–	–	0.98	[0.26,	3.79]	.982

*Note*. Abbreviations: ASC-H, atypical squamous cells cannot exclude high grade; ASC-US, atypical squamous cells of undetermined significance; HSIL, high-grade intraepithelial lesion; LSIL, low-grade intraepithelial lesion; NILM, negative for intraepithelial lesion or malignancy; OR = Odds Ratio

## Data Availability

The datasets generated and analyzed during this study are not publicly available due to restriction to only investigators included on the study protocol decided by the Institutional Review Board of the University of Mississippi Medical Center. Summary data can be made available from the corresponding author upon request.
